# Supplementary material for: Trends and determinants of clustering for non-communicable disease risk factors in women of reproductive age in Nepal
Source: PLoS One. 2024 Oct 2;19(10):e0309322. doi: 10.1371/journal.pone.0309322 (PMC11446422; doi:10.1371/journal.pone.0309322)
Supplement: S1 Table — (PDF) [file pone.0309322.s001.pdf]

Table S1: Output of independent sample proportion test (Z test) for NCD risk factor among women of reproductive age (15-49 years) between NDHS 2016 and 2022

Variable	Current Smoking		P value	Overweight/Obesity		P value	Hypertension		P value
**Provinces**	**2016(N=6,069) ***	**2022(N=6,980) ***		**2016(N=6,069)***	**2022(N=6,980) ***		**2016(N=6,067) ***	**2022(N=3,765) ***	
Koshi	3.3	3.6	0.700	27.4	28.3	0.63	6.50	13.4	**<0.001**
Madhesh	2.3	0.6	**<0.001**	10.8	18.5	**<0.001**	4.30	5.5	0.220
Bagmati	8.4	6.2	**<0.050**	34.8	44.3	**<0.001**	4.90	11.6	**<0.001**
Gandaki	6.1	2.6	**<0.010**	31.6	40.3	**<0.001**	6.50	12	**<0.010**
Lumbini	4.6	3.3	0.115	18.5	28.2	**<0.001**	5.20	8.7	**<0.010**
Karnali	14.1	9.1	**<0.001**	10.3	18.8	**<0.001**	4.80	8.2	0.097
Sudurpaschim	10.8	4.2	**<0.050**	9	14.7	**<0.010**	3.10	6.3	**<0.050**
National	6.01	3.86	**<0.001**	22.15	29.02	**<0.001**	5.09	9.57	**<0.001**

*Note: * weighted sample size for the risk factor, bold letter denotes significant difference in percentage point of risk factor between two round of surveys (NDHS 2016 and 2022)*
