# Supplementary material for: Trends and determinants of clustering for non-communicable disease risk factors in women of reproductive age in Nepal
Source: PLoS One. 2024 Oct 2;19(10):e0309322. doi: 10.1371/journal.pone.0309322 (PMC11446422; doi:10.1371/journal.pone.0309322)
Supplement: Attachment — Submitted filename: Final_NCD Manuscript_30.5.2024_BKS_feedback.docx [file pone.0309322.s003.docx]

**Patterns of Non-Communicable Diseases Risk Factors Clustering Among Women of Reproductive Age in Nepal: Insights From a Nationally Representative Households Survey**

Barun Kumar Singh MMed. Stat^1*^, Shiva Raj Mishra PhD ^2,3^, Resham B Khatri PhD ^4^

^1^ Health Nutrition Education and Agriculture Research Development, Saptari, Nepal

^2^ Nepal Development Society, Bharatpur, Chitwan, Nepal

^3^ Faculty of Medicine and Health, University of Sydney, Sydney, Australia

^4^School of Public Health, University of Queensland, Brisbane, Australia

*Corresponding author:

Barun Kumar Singh, MMed Stat

Health Nutrition Education and Agriculture Research Development, Saptari, Nepal

Email: [barun272@gmail.com](mailto:barun272@gmail.com)

Words:8167
Figure: 2

Tables: 4

**ABSTRACT**

**Background**

Understanding the clustering of two or more risk factors, such as smoking, overweight/obesity, and hypertension, among women of reproductive age could facilitate the design and implementation of strategies to address the growing risk factor burden in Nepal. We aimed to assess the trends of prevalence and associated factors with smoking status, overweight/obesity, and hypertension among Nepalese women of reproductive age (15-49 years).

**Methods**

We examined the 2022 Nepal Demographic and Health Survey (NDHS) data to assess NCD risk factors clustering and compared that with NDHS 2016 to explore the trends in NCD risk factors between 2016 and 2022. A sample of 6,957 women aged 15–49 years were included to determine the factors related to smoking and obesity/overweight, while 3,749 women of reproductive age were included to examine hypertension status in 2022. Poisson regression models with robust sandwich variance estimates were used to estimate the adjusted prevalence ratio (APR) for outcome variables and the clustering of NCD risk factors across various socio-demographic variables.

**Results**

The average age of participants was 30 (SD = 9.7) years. The prevalence rates for smoking, overweight/obesity, and hypertension were 3.8%, 29.2%, and 9.6%, respectively. More than one-fourth of the participants (28.7%) had one NCD risk factor, while 6.5% had two such risk factors. Women aged 40–49 years were more likely to have multiple NCD risk factors than those aged 15–29 (APR: 3.19; 95% CI: 2.68–3.80). Those in the richest wealth quintile (APR: 1.52; 95% CI: 1.24–1.85), as well as married (APR: 3.02; 95% CI: 2.43–3.76) and widowed/divorced (APR: 2.85; 95% CI: 2.14–3.80) individuals, were more likely to experience multiple NCD risk factors. Participants in the relatively affluent Koshi province (APR: 1.74; 95% CI: 1.41–2.15) had more NCD risk factors than those in the Sudurpaschim province. Additionally, women belonging to Hill Janajatis (APR: 1.44; 95% CI: 1.21–1.72) and Dalit (APR: 1.42; 95% CI: 1.15–1.75) ethnic groups were more likely to exhibit risk factors for NCDs.

**Conclusions**

The study found that NCD risk factors are more common among women in older age groups, those who are currently married or widowed/divorced/separated, and individuals from the wealthiest socioeconomic groups. A higher burden of risk factors underscores the importance of targeted public health interventions, particularly among higher socio-economic groups and those of affluent regions.

**Keywords:** Non-Communicable Diseases, Smoking, Overweight, Obesity, Hypertension, Reproductive Age Women

**INTRODUCTION**

Non-communicable diseases (NCDs) represent a leading cause of global mortality and disability (1). NCDs contribute to 41 million deaths each year; 77% of deaths occur in Low and Middle-Income Countries (LMICs). In Nepal, NCDs were responsible for an estimated 117,300 deaths in 2019, comprising 66% of total mortality (2). Further, epidemiological studies have shown an increasing trend for NCDs and their risk factors over the last two decades, such as hypertension prevalence increased from 21% to 25% between 2007 and 2019 (3). Similarly, the prevalence of Diabetes Mellitus (DM) increased from 3.6% in 2013 to 8% in 2019 (3-5).

Nepal’s epidemiological transition is amid gradual economic growth and increased rural-to-urban migration (6, 7). Consequently, most of the population is adopting a sedentary lifestyle, influenced by changes in dietary habits, improved accessibility and preference for unhealthy foods, decreased levels of physical activity, and alarming rates of smoking and alcohol consumption (3, 8-10). Women face an elevated risk of developing NCDs as they are more prone to experiencing a combination of behavioural and metabolic risk factors (11-14). Hypertension in women can affect the normal functioning of the reproductive system, leading to menstrual irregularities, diminished fertility, and maternal mortality (15, 16). Similarly, being overweight and obese can increase the risk of diabetes, cardiovascular diseases(CVDs), cancer, and mortality, emphasizing the urgent need for lifestyle changes (17). According to the World Health Organization (WHO), 95% of all maternal deaths occurred in LMICs in 2020, and most of the maternal deaths were due to pre-existing medical conditions or clustering of both behavioural and metabolic risk factors (18). More importantly, women possessing the risk factors for NCDs have negative repercussions on their reproductive health and their offspring, leading to an intergenerational cycle of NCD risk (19, 20). Such NCD risk factors could be higher among women in Nepal due to a higher rate of undernutrition, overnutrition, and hypertension among women during pregnancy (21).

Nepal's health system has addressed the growing burden of NCD risk factors through a multifaceted approach, including national policies and regulations like the multisectoral action plans (2016-2020 and 2021-2025) (22, 23) and the Tobacco Products Control and Regulation Act (2010) (24) aligned with WHO Framework Convention on Tobacco Control(FCTC)(25). The National Health Education, Information, and Communication Centre (NHEICC) conducts tobacco-specific advocacy and awareness activities (26). Health promotion activities targeting modifiable risk factors such as unhealthy diet/nutrition and physical activities are ongoing in primary healthcare settings (27).

NCD screening and management have been integrated into primary health services across 77 districts through the Package of Essential Non-Communicable Diseases (PEN) packages (26). However, challenges such as a lack of targeted approach for health promotion interventions, scarce healthcare resources, and inconsistent regulation enforcement require ongoing efforts to strengthen the health system's capacity to manage the increasing burden of NCD risk factors (7, 25, 27-29). More importantly, women have been reported to be vulnerable to obesity, physical inactivity, and unhealthy behaviours due to gender norms and social determinants (30).

Women’s NCD risk has remained under-recognized due to low awareness, screening, and diagnosis compared to men in low-income settings like Nepal (31, 32). To successfully fill this gap, it is imperative to take a comprehensive and structured approach to assess women’s NCD risk factors, which will help in designing and implementing targeted interventions to address the prevention and control of NCDs. Previous studies were unable to provide disaggregated results by sex (4, 7, 33-35), focused on standalone risk factors such as Diabetes Mellitus (36) and hypertension (37-39), and included specific geographical areas (31, 33, 35, 38) and age groups (40, 41) This study, therefore, aimed to i.) analyze the prevalence and trends of NCD risk factors between the two rounds of NDHS 2016 and 2022. ii.) investigate the socio-demographic determinants of NCD risk factor clustering among Nepalese women of reproductive age (15-49 years).

**METHODS**

**Study Population and Data Sources**

The prevalence trend of risk factors was estimated using two nationally representative NDHS data (21, 42). To identify the prevalence and significant determinants of NCD risk factors among women, this study used the NDHS 2022 data set. The NDHS 2016 and 2022 report describes the detailed sampling strategy and participants (21, 42). Data were extracted for all eligible women (n = 6,957) aged 15–49 years and included in the final model for overweight/obesity and current smoking status. Blood pressure was measured among 3970 eligible women, of which 221 were either pregnant or recently delivered, which was excluded from the analysis. This resulted in the final model for hypertension and clustered risk factors (n=3749).

**Outcome Variables**

The outcome variables for this study considered the three risk factors associated with NCDs: smoking, overweight and obesity, and hypertension. These specific NCD risk factors were extracted from the NDHS 2022 survey dataset. Furthermore, we defined the clustering of these three risk factors and examined their prevalence and the associated factors.

**Current Smoking:** Within the NDHS survey, participants' current smoking status was documented as a dichotomous response.

**Overweight and Obesity:** Women's weight and height were measured following the DHS VIII biomarker manual (43). Women who were pregnant during the survey visit or had given birth within the two months prior were excluded from the study. Body mass index (BMI) was calculated by dividing weight in kilograms by the square of height in meters. Women with a BMI between 25.0 and 29.9 kg/m^2^ were considered overweight, whereas individuals with a BMI greater than or equal to 30.0 kg/m^2^ were classified as obese (21)We merged the overweight and obese categories into a single binary variable, defining an individual as overweight or obese if their BMI was 25.0 kg/m^2^ or higher.

**Hypertension:** Blood pressure was measured for all participants aged 15 and above within a subset comprising about one-fourth of the households. Individuals eligible for blood pressure measurements were contacted and provided with instructions on the procedure—those who consented provided blood pressure measurements. An automatic device with separate cuffs was used for blood pressure monitoring. Three blood pressure readings were taken at intervals of approximately 10 minutes. Typically, the average of the second and third readings was used to represent the participants’ blood pressure. Hypertension was diagnosed if the average systolic blood pressure reading was 140 mmHg or higher, the average diastolic blood pressure was 90 mmHg or higher, or if individuals were taking antihypertensive medication at the time of the survey (44, 45).

**Risk Factors Clustering:** The clustering of NCD risk factors was defined as the presence of multiple risk factors in each participant. Assessment of the clustering of risk factors involved counting the risk factors from smoking, overweight/obesity, or hypertension. Thus, their presence was categorized within a range of 0 to 3.

**Explanatory Variables**

We considered socio-demographic variables related to smoking, overweight/obesity, and hypertension. These included the respondents’ age(15-29, 30-39, and 40-49 years), education attainment(no education, primary education, some secondary education, SLC or higher education), marital status(married, never married, widowed/divorces/separated), residence (rural, urban), place of residence(rural, urban), provinces(Koshi, Madhesh, Bagmati, Gandaki, Lumbini, Karnali, Sudurpaschim), occupational status (not working, service, agriculture/self-employed, and manual), Caste/Ethnicity (Brahmin hill, Chhetri hill, Terai caste, Dalit, Hill Janajati, Terai Janajati, Muslim), wealth index(poorest, poorer, middle, richer and richest). The wealth index is derived from a composite measure of a household's living standard. This index is computed using readily obtainable data on a household's ownership of specific assets, housing construction materials, and access to water and sanitation facilities. Subsequently, national wealth quintiles were constructed by assigning the household score to each usual household member, ranking individuals in the household population based on their score, and dividing the distribution into five equal categories, each representing 20% of the population (46).

**Statistical Analysis**

The sociodemographic characteristics of the women involved in the study were presented using mean values and weighted percentages. We calculated the crude prevalence of smoking, overweight/obesity, and hypertension by taking the complex survey design and sampling weights into account. The association between categorical variables was assessed using the chi-square test. Variables with a chi-square test p-value below 0.25 were included in the multivariable models. Modified Poisson regression models with a robust sandwich variance estimate were used to identify factors associated with NCD risk factors (smoking, overweight/obesity, and hypertension), producing adjusted prevalence ratios (APRs) and corresponding 95% confidence intervals (CIs)(11). Variables such as ecological zones were excluded after a multicollinearity check revealed a variance inflation factor (VIF) of ≥ 5. This choice of the Poisson regression model was made due to concerns that logistic regression, commonly used in cross-sectional studies with binary outcomes, tends to estimate the relative risk poorly, as noted in previous research (47-49). Secondly, a modified Poisson regression model with a robust sandwich variance estimate was used as an alternative to binary logistic regression, considering the prevalence of binary outcome was ≥10 % (11, 50, 51). Finally, the number of risk factors present with the individual was aggregated, ranging from 0 to 3, to examine the clustering of risk factors. This analysis used the modified Poisson regression model with count data as an outcome and a robust sandwich variance estimate to consider the underlying assumption of the conventional Poisson regression model (52). We used STATA 18 software to perform the statistical analysis by deploying two-tailed tests with statistical significance defined as a p-value of less than 0.05. Provided that the NDHS 2022 survey adopted a two-stage stratified cluster sampling technique, the analysis used recommended sample weights (21). Further, we adjusted the clustering effect using the “svy” command in STATA.

**RESULTS**

**Trends in the Prevalence of NCD Risk Factors among Reproductive-Aged Women Over the Surveys.**

**Figure 1** shows a significant decreasing trend in smoking prevalence among women in all provinces except for Koshi province over the NDHS survey years 2016-2022. In contrast, the prevalence of overweight and obesity among women rose significantly across all the provinces, excluding Koshi provinces in the same period. Likewise, the prevalence of hypertension also increased significantly in all provinces except Karnali and Madhesh province. The prevalence of current smoking ranged from 2.3% in Madhesh to 14.1% in Karnali in 2016, which significantly declined in 2022, ranging from 0.6% in Madhesh to 9.1% in Karnali. Overweight and obesity rates increased nationally from 22.2% in 2016 to 29.0% in 2022, notably rising in Bagmati up to 44.3% and Gandaki up to 40.3%. Similarly, hypertension prevalence nearly doubled nationally from 5.0% to 9.6% between 2016 and 2022, with significant increases observed in Koshi (6.5% to 13.4%), Bagmati (4.9% to 11.6%), Gandaki (6.5% to 12.0%), Lumbini (3.1% to 6.3%), and Sudurpaschim (3.1% to 6.3%).

…………………………………….insert figure 1 here……………………………………….

**Figure 1: Province and National Trend of NCD Risk Factors Among Reproductive Age Women Over the Surveys**

#### **Characteristics of the Study Participants from NDHS 2022**

**Table 1** depicts the sociodemographic characteristics of the study participants (n=6957) for current smoking and overweight/obesity, hypertension, and the clustering of NCD risk factors (n=3749). The mean age of study participants was 30 years (SD=9.7). Almost two-thirds of them were from urban. Approximately half of them were between 15 and 29 years old. In terms of educational attainment, one-fourth of them did not have any formal education. One-sixth of them had qualifications above the secondary level. Based on the wealth quintile, the richest represented 22.6% of the participants. Approximately half of the participants were engaged in agriculture or self-employment.

**Table 1: Sociodemographic Characteristics of Reproductive Aged Women (15-49 years).**

| **Variables** | **Unweighted Count** | **Weighted %/Mean (SD)** | **Unweighted Count** | **Weighted %/Mean (SD)** |
| --- | --- | --- | --- | --- |
|  | **n=6957*** |  | **n=3749*** |  |
| **Age (Years)** |  | **30.0(9.7)** |  | **30.0(9.7)** |
| **Body Mass Index (Kg/Sq Meter)** |  | **23.0(4.4)** |  | **23.0(4.4)** |
| **Place of Residence** |  |  |  |  |
| Urban | 3,751 | 68.6 | 2,026 | 68.9 |
| Rural | 3,206 | 31.4 | 1,723 | 31.1 |
| **Ecological Zone** |  |  |  |  |
| Mountain | 566 | 5.1 | 295 | 4.9 |
| Hill | 3,146 | 40.7 | 1,712 | 41.1 |
| Terai | 3,245 | 54.2 | 1,742 | 54 |
| **Province** |  |  |  |  |
| Koshi | 1,054 | 16.9 | 564 | 17 |
| Madhesh | 1,126 | 19.4 | 617 | 19.7 |
| Bagmati | 1,008 | 21.4 | 545 | 21.6 |
| Gandaki | 803 | 9.6 | 431 | 9.5 |
| Lumbini | 1,070 | 18 | 556 | 17.3 |
| Karnali | 911 | 6 | 490 | 6 |
| Sudurpaschim | 985 | 8.6 | 546 | 8.8 |
| **Native Language** |  |  |  |  |
| Nepali | 5,423 | 75.4 | 2,933 | 76.1 |
| Maithali | 793 | 13.6 | 426 | 13.4 |
| Bhojpuri | 343 | 5.6 | 188 | 5.6 |
| Other | 398 | 5.4 | 202 | 4.9 |
| **Marital Status** |  |  |  |  |
| Never Married | 1,527 | 22.3 | 833 | 22.9 |
| Married or Living together | 5,205 | 74.6 | 2,790 | 73.8 |
| Widow/Divorced/Separated | 225 | 3.1 | 126 | 3.3 |
| **Women's Occupational Status** |  |  |  |  |
| Not Working | 1,637 | 27.4 | 891 | 27.1 |
| Services | 937 | 16.1 | 513 | 16.8 |
| Agriculture-Self employed | 3,836 | 48.3 | 2,040 | 47.5 |
| Manual | 547 | 8.3 | 305 | 8.5 |
| **Women's Education** |  |  |  |  |
| No education | 1,904 | 25.5 | 1,036 | 25.9 |
| Primary | 2,224 | 31.3 | 1,181 | 30.5 |
| Some secondary | 1,782 | 25.9 | 966 | 25.6 |
| SLC and above | 1,047 | 17.3 | 566 | 18 |
| **Women's Age** |  |  |  |  |
| 15-29 yrs | 3,526 | 50.2 | 1,875 | 49.2 |
| 30-39 yrs | 1,966 | 29.1 | 1,083 | 30 |
| 40-49 yrs | 1,465 | 20.7 | 791 | 20.8 |
| **Caste/Ethnicity** |  |  |  |  |
| Brahmin Hill | 655 | 10.2 | 360 | 10.1 |
| Chhetri Hill | 1,642 | 18.3 | 887 | 18.8 |
| Terai Caste | 886 | 15.6 | 462 | 15 |
| Dalit | 1,158 | 15 | 636 | 15.1 |
| Hill Janajati | 1,698 | 26.3 | 913 | 26.4 |
| Terai Janajati | 697 | 10.5 | 375 | 10.6 |
| Muslim | 221 | 4.1 | 116 | 4 |
| **Wealth Index Combined** |  |  |  |  |
| Poorest | 1,837 | 17.3 | 993 | 17.3 |
| Poorer | 1,478 | 20.1 | 817 | 20.6 |
| Middle | 1,404 | 20.2 | 720 | 19 |
| Richer | 1,198 | 19.9 | 657 | 20.4 |
| Richest | 1,040 | 22.6 | 562 | 22.6 |

**Table 2** depicts the prevalence of risk factors for NCDs among reproductive-aged women. The overall prevalence of smoking was 3.8% among the study participants. Smoking was more prevalent among women from the Mountainous zone (8.4%), Karnali province (9.1%), and Nepalese native speaking (4.6%). Likewise, women with widow/divorced/separated status (9.6%), age group of 40-49 years (9.7%), manual working occupational status (7.1%), no formal education (9.0%), Dalit ethnicity (5.5%) had a relatively higher prevalence of smoking compared to their counterparts (p<0.001). Relative to other risk factors, overweight and obesity were common among the participants (29.2%).

More than four out of ten (44.4%) of the participants between 40-49 years were either overweight or obese (p<0.001). Similarly, overweight and obesity were more prevalent among hilly zone residents (35.8%), Bagmati province residents (44.3%), Nepali native speakers (33.4%), widow/divorced/separated participants (36.5%), service holders (45.6%), higher educated (32.4%), hill Janajati residents (42.2%), the richest (46.7%) (p<0.001). In terms of hypertension, 9.3% of women were suffering from hypertension. Women aged 40-49 years had the highest prevalence of hypertension (24.8%) (p<0.001). In addition to this, the prevalence of hypertension was higher among women who were not living with husbands or widowed (24%), with no education (14.5%), Nepalese native speakers (11%), and from hill Janajati families (13.2%) (p<0.001)**.**

**Table 2: Prevalence of Non-communicable Disease Risk Factors Among Reproductive-Aged Women (15-49 years)*.**

|  | **Current Smoking** | | **Overweight and Obese** | | **Hypertension** | |
| --- | --- | --- | --- | --- | --- | --- |
| **Variables** | **Weighted n** | **Prevalence, % (95% CI)** | **Weighted n** | **Prevalence- % (95% CI)** | **Weighted n** | **Prevalence, % (95% CI)** |
| **Total** | **6,980** | **3.8(3.3-4.4)** | **6,980** | **29.2(27.6-30.9)** | **3,765** | **9.6(8.4-10.8)** |
| **Place of Residence** |  | Yes (%) |  | Yes (%) |  | Yes (%) |
| Urban | 4,791 | 3.2(2.6-3.9) | 4,791 | 32.6(30.5-34.9) | 2,595 | 9.9(8.4-11.6) |
| Rural | 2,189 | 5.3(4.4-6.3) | 2,189 | 21.7(19.8-23.6) | 1,170 | 8.9(7.3-10.7) |
|  | p<0.01 |  | p<0.01 |  | p=0.377 |  |
| **Ecological Zone** |  |  |  |  |  |  |
| Mountain | 356 | 8.4(5.8-12.2) | 356 | 20.9(14.1-29.7) | 185 | 11(5.5-21.0) |
| Hill | 2,839 | 5.1(4.1-6.2) | 2,839 | 35.8(33.2-38.4) | 1,548 | 11.7(9.9-13.8) |
| Terai | 3,785 | 2.5(1.9-3.2) | 3,785 | 25.1(22.6-27.7) | 2,032 | 7.8(6.4-9.4) |
|  | p<0.001 |  | p<0.001 |  | p<0.05 |  |
| **Provinces** |  |  |  |  |  |  |
| Koshi | 1,183 | 3.6(2.5-5.3) | 1,183 | 28.3(24.8-32.1) | 640 | 13.4(10.2-17.3) |
| Madhesh | 1,356 | 0.6(0.3-1.3) | 1,356 | 18.5(15.2-22.2) | 743 | 5.5(3.8-7.9) |
| Bagmati | 1,494 | 6.2(4.7-8.3) | 1,494 | 44.3(40.5-48.2) | 812 | 11.6(8.7-15.3) |
| Gandaki | 667 | 2.6(1.6-4.1) | 667 | 40.3(36.0-44.8) | 359 | 12(8.9-15.9) |
| Lumbini | 1,259 | 3.3(2.2-5.1) | 1,259 | 28.2(24.1-32.8) | 652 | 8.7(6.3-11.8) |
| Karnali | 421 | 9.1(7.2-11.5) | 421 | 18.8(14.8-23.5) | 226 | 8.2(5.6-11.9) |
| Sudurpaschim | 600 | 4.2(3.0-5.9) | 600 | 14.7(11.7-18.3) | 332 | 6.3(4.5-8.7) |
|  | p<0.001 |  | p<0.001 |  | p<0.001 |  |
| **Languages** |  |  |  |  |  |  |
| Nepali | 5,264 | 4.6(4.0-5.4) | 5,264 | 33.4(31.5-35.3) | 2,863 | 11(9.6-12.6) |
| Maithali | 948 | 0.9(0.4-1.8) | 948 | 15(11.8-18.9) | 505 | 5.5(3.6-8.4) |
| Bhojpuri | 388 | 0.1(0.0-1.1) | 388 | 15.4(12.2-19.3) | 210 | 2.1(0.8-5.3) |
| Other | 380 | 4.1(2.3-7.2) | 380 | 20.9(16.5-26.1) | 186 | 6.7(4.1-10.7) |
|  | p<0.001 |  | p<0.001 |  | p<0.001 |  |
| **Marital Status** |  |  |  |  |  |  |
| Never Married | 1,556 | 1.1(0.6-2.0) | 1,556 | 8.7(6.8-11.2) | 862 | 3.9(2.6-6.0) |
| Married or Living together | 5,208 | 4.4(3.8-5.2) | 5,208 | 35(33.0-37.0) | 2,779 | 10.7(9.3-12.2) |
| Widow/Divorced/Separated | 216 | 9.6(6.2-14.5) | 216 | 36.5(29.1-44.7) | 123 | 24(16.4-33.6) |
|  | p<0.001 |  | p<0.001 |  | p<0.001 |  |
| **Women's Occupational Status** |  |  |  |  |  |  |
| Not Working | 1,910 | 0.9(0.5-1.6) | 1,910 | 26.9(24.0-30.0) | 1,020 | 7(5.1-9.5) |
| Services | 1,121 | 2.3(1.4-3.8) | 1,121 | 45.6(41.5-49.8) | 634 | 12.1(9.2-15.8) |
| Agriculture-Self employed | 3,371 | 5.5(4.7-6.4) | 3,371 | 24.6(22.7-26.5) | 1,789 | 9.3(7.8-11.0) |
| Manual | 578 | 7.1(4.8-10.5) | 578 | 31.9(27.0-37.3) | 321 | 14.5(10.5-19.7) |
|  | p<0.001 |  | p<0.001 |  | p<0.01 |  |
| **Women's Education** |  |  |  |  |  |  |
| No education | 1,779 | 9(7.6-10.5) | 1,779 | 26.4(23.9-29.1) | 975 | 14.5(12.0-17.4) |
| Primary | 2,183 | 3.2(2.3-4.4) | 2,183 | 30.5(28.0-33.1) | 1,149 | 9.4(7.7-11.4) |
| Some secondary | 1,809 | 1.3(0.8-2.4) | 1,809 | 28.3(25.5-31.2) | 965 | 7.1(5.5-9.3) |
| SLC and above | 1,209 | 1.2(0.6-2.4) | 1,209 | 32.4(28.8-36.1) | 676 | 6.3(4.2-9.3) |
|  | p<0.001 |  | p<0.001 |  | p<0.001 |  |
| **Women's Age** |  |  |  |  |  |  |
| 15-29 yrs | 3,505 | 1.4(1.0-2.1) | 3,505 | 15.9(14.2-17.7) | 1,853 | 2.8(2.0-3.9) |
| 30-39 yrs | 2,029 | 3.8(3.0-4.8) | 2,029 | 41.7(38.8-44.6) | 1,128 | 10.1(8.1-12.5) |
| 40-49 yrs | 1,446 | 9.7(8.2-11.6) | 1,446 | 44.1(40.9-47.3) | 783 | 24.8(21.1-28.9) |
|  | p<0.001 |  | p<0.001 |  | p<0.001 |  |
| **Caste/Ethnicity** |  |  |  |  |  |  |
| Brahmin Hill | 713 | 1.0(0.5-2.2) | 713 | 35.5(30.8-40.6) | 381 | 9.2(5.9-14.1) |
| Chhetri Hill | 1,276 | 5.0(4.0-6.2) | 1,276 | 26.6(23.2-30.3) | 707 | 9.4(7.3-12.2) |
| Terai Caste | 1,088 | 1.0(0.5-2.0) | 1,088 | 19.4(16.6-22.5) | 566 | 4.7(3.1-6.9) |
| Dalit | 1,050 | 5.5(4.0-7.4) | 1,050 | 25.9(22.4-29.8) | 567 | 10.5(8.0-13.6) |
| Hill Janajati | 1,833 | 5.4(4.2-7.0) | 1,833 | 42.2(39.4-45.0) | 994 | 13.2(10.6-16.3) |
| Terai Janajati | 732 | 3.9(2.6-5.8) | 732 | 16.9(13.4-21.0) | 400 | 8.4(5.9-11.8) |
| Muslim | 287 | 0.2(0.0-1.7) | 287 | 22.5(16.7-29.5) | 151 | 5.5(2.5-11.9) |
|  | p<0.001 |  | p<0.001 |  | p<0.001 |  |
| **Wealth Index Combined** |  |  |  |  |  |  |
| Poorest | 1,207 | 8.1(6.8-9.7) | 1,207 | 15.9(13.9-18.1) | 650 | 9.8(7.5-12.7) |
| Poorer | 1,400 | 3.9(3.0-5.1) | 1,400 | 20.2(17.8-22.8) | 778 | 7.2(5.4-9.4) |
| Middle | 1,411 | 2.6(1.8-3.8) | 1,411 | 23.5(21.1-26.2) | 717 | 8(6.1-10.4) |
| Richer | 1,387 | 3.4(2.3-5.0) | 1,387 | 35.8(32.3-39.5) | 769 | 12.4(9.9-15.5) |
| Richest | 1,574 | 2(1.2-3.1) | 1,574 | 46.7(42.8-50.6) | 852 | 10.4(7.4-14.3) |
|  | p<0.001 |  | p<0.001 |  | p=0.05 |  |

**Note: *excludes reproductive-aged women aged 15-49 years with pregnancy and post-partum status (up to 2 months)**

Furthermore, we assessed the prevalence of risk factors for NCDs risk factors by the number of factors across different age groups (**Figure 2**). Over one-fourth of the participants (28.71%) had one NCD risk factor, and 6.56% showcased the prevalence of two NCD risk factors.

…………………………………..insert figure 2 here……………..…………………………..

**Figure 2:** **Prevalence of a number of non-communicable disease risk factors among reproductive-aged women.**

**Table 3** depicts the adjusted association between sociodemographic characteristics and NCD risk factors derived from the modified Poisson regression models. Women’s age was strongly associated with current smoking for NCDs. There was a higher prevalence of smoking among women aged 40-49 years (APR:1.77; 95% CI:1.79-5.01) than women aged 15-29 years. The prevalence ratio of smoking was three times higher among women with no education (APR: 3.06; 95% CI1.50-6.25) compared to those women with a School Leaving Certificate (SLC) or a higher level of education. The prevalence ratio of the women from Bagmati (APR:2.54; 95% CI:1.59-4.04) and Karnali (APR:1.88; 95% CI:1.30-2.73) was relatively higher than women from Sudurpaschim. Compared to those who were not employed, those whose occupations involved agriculture or self-employment (APR:2.92; 95% CI:1.63-5.24) and manual work (APR: 3.60; 95% CI: 1.88-6.90) had a higher prevalence ratio for current smoking. In terms of caste/ethnicity, the prevalence ratio for women from Chhetri (APR:3.13; 95% CI:1.32-7.42), Hill Janajati (APR: 3.25; 95% CI:1.38-7.68), and Terai Janajati (APR:3.12; 95% CI:1.18-8.27) was about three folds higher relative to women from Brahmin hill. Similarly, Dalit women were 4.22(APR: 4.22; 95% CI:1.75-10.20) times more likely to be current smokers relative to Brahmin Hill women.

The prevalence ratio for overweight and obesity in the age 30-39 years (APR:1.80; 95% CI: 1.60-2.02) and 40-49 years (APR:2.01; 95% CI:1.77-2.29) was significantly higher than 15-29 years. Married women (APR:3.02; 95% CI:2.43-3.76) and widowed/divorced/separated women (APR:2.85; 95% CI:2.14-3.80) illustrated a higher prevalence of overweight/obesity compared to the never-married women. Overweight and obesity were almost 1.5 times higher among the women from Koshi, Madhesh, and Lumbini than the women from Sudurpaschim. Likewise, women from Bagmati (APR:1.69; 95% CI:1.40-2.04), Karnali (APR:1.28; 95% CI: 1.03-1.59), and Gandaki (APR:1.60; 95% CI:1.33-1.94) had also higher prevalence ratio for overweight/obesity compared to Sudurpaschim. Moreover, individuals who were overweight and obese were more likely to belong to the richest (APR:2.23; 95% CI:1.86-2.67), richer (APR:2.02; 95% CI:1.71-2.37), middle (APR:1.58; 95% CI:1.34 - 1.87), and poorer (APR: 1.36; 95% CI:1.15-1.60) compared to poorest women.

Similarly, the prevalence of hypertension was significantly higher in the 30-39 years (APR: 3.42; 95% CI:2.21-5.27) and 40-49 years (APR: 8.47; 95% CI:5.47-13.11) age groups compared to the younger women. Hypertension was 2.2 (APR: 2.20; 95% CI:1.41 – 3.33) times higher among the women from Koshi than those from Sudurpaschim.

**Table 3: Association between sociodemographic characteristics and NCD risk factors**

| **Variables** | **Current Smoking, APR (95% CI)** | **Overweight and Obesity, APR (95% CI)** | **Hypertension, APR (95% CI)** |
| --- | --- | --- | --- |
| **Place of Residence** |  |  |  |
| Urban | 1.00 | 1.00 | 1.00 |
| Rural | 0.92(0.72 - 1.17) | 1.08(0.99 - 1.19) | 1.04(0.82 - 1.30) |
| **Province** |  |  |  |
| Koshi | 1.29(0.80 - 2.09) | 1.52(1.26 - 1.84) *** | 2.20(1.41 - 3.44)*** |
| Madhesh | 0.53(0.18 - 1.59) | 1.54(1.21 - 1.96) *** | 1.74(0.92 - 3.29) |
| Bagmati | 2.54(1.59 - 4.04) *** | 1.69(1.40 - 2.04) *** | 1.54(0.95 - 2.51) |
| Gandaki | 0.91(0.52 - 1.59) | 1.60(1.33 - 1.94) *** | 1.59(0.97 - 2.61) |
| Lumbini | 1.06(0.67 - 1.69) | 1.50(1.25 - 1.80) *** | 1.46(0.92 - 2.34) |
| Karnali | 1.88(1.30 - 2.73) *** | 1.28(1.03 - 1.59) * | 1.28(0.78 - 2.09) |
| Sudurpaschim | 1.00 | 1.00 | Reference |
| **Native Language** |  |  |  |
| Nepali | 13.10(1.60 - 107.01) * | 1.55(1.14 - 2.11) ** | 3.72(1.30 - 10.65) * |
| Maithali | 5.55(0.68 - 45.18) | 0.88(0.64 - 1.20) | 2.34(0.80 - 6.85) |
| Bhojpuri | Reference | Reference | Reference |
| Other | 14.47(1.69 - 123.95) * | 1.37(0.96 - 1.95) | 3.30(1.01 - 10.81) * |
| **Marital Status** |  |  |  |
| Never Married | 1.00 | 1.00 | 1.00 |
| Married or Living together | 1.18(0.56 - 2.48) | 3.02(2.43 - 3.76) *** | 0.87(0.53 - 1.42) |
| Widow/Divorced/Separated | 1.47(0.64 - 3.34) | 2.85(2.14 - 3.80) *** | 1.10(0.60 - 2.02) |
| **Women's Occupational Status** |  |  |  |
| Not Working | 1.00 | 1.00 | 1.00 |
| Services | 2.08(0.94 - 4.62) | 1.13(1.01 - 1.27) * | 1.17(0.81 - 1.69) |
| Agriculture-Self employed | 2.92(1.63 - 5.24) *** | 0.94(0.84 - 1.05) | 0.84(0.61 - 1.17) |
| Manual | 3.60(1.88 - 6.90) *** | 0.93(0.79 - 1.08) | 1.34(0.90 - 2.00) |
| **Women's Education** |  |  |  |
| No education | 3.06(1.50 - 6.25) ** | 0.86(0.73 - 1.00) | 1.19(0.74 - 1.92) |
| Primary | 1.59(0.76 - 3.34) | 1.02(0.90 - 1.17) | 1.15(0.75 - 1.75) |
| Some secondary | 0.93(0.42 - 2.07) | 1.03(0.91 - 1.17) | 1.01(0.65 - 1.55) |
| SLC and above | 1.00 | 1.00 | 1.00 |
| **Women's Age** |  |  |  |
| 15-29 yrs | 1.00 | 1.00 | 1.00 |
| 30-39 yrs | 1.53(0.92 - 2.54) | 1.80(1.60 - 2.02) *** | 3.42(2.21 - 5.27) *** |
| 40-49 | 2.99(1.79 - 5.01) *** | 2.01(1.77 - 2.29) *** | 8.47(5.47 - 13.11) *** |
| **Caste/Ethnicity** |  |  |  |
| Brahmin Hill | 1.00 | 1.00 | 1.00 |
| Chhetri Hill | 3.13(1.32 - 7.42) ** | 1.07(0.92 - 1.25) | 1.16(0.73 - 1.84) |
| Terai Caste | 2.53(0.68 - 9.36) | 0.99(0.81 - 1.20) | 0.80(0.44 - 1.44) |
| Dalit | 4.22(1.75 - 10.20) ** | 1.33(1.13 - 1.57) *** | 1.45(0.90 - 2.33) |
| Hill Janajati | 3.25(1.38 - 7.68) ** | 1.44(1.26 - 1.64) *** | 1.37(0.90 - 2.08) |
| Terai Janajati | 3.12(1.18 - 8.27) * | 0.77(0.62 - 0.96) * | 1.06(0.63 - 1.79) |
| Muslim | 0.45(0.05 - 3.90) | 1.30(0.98 - 1.73) | 0.90(0.40 - 2.03) |
| **Wealth Index Combined** |  |  |  |
| Poorest | 1.00 | Refe | Reference |
| Poorer | 0.68(0.49 - 0.93)* | 1.36(1.15 - 1.60)*** | 0.70(0.49 - 1.00) |
| Middle | 0.54(0.36 - 0.82)** | 1.58(1.34 - 1.87)*** | 0.84(0.58 - 1.23) |
| Richer | 0.77(0.50 - 1.18) | 2.02(1.71 - 2.37)*** | 1.16(0.81 - 1.65) |
| Richest | 0.57(0.33 - 0.97)* | 2.23(1.86 - 2.67)*** | 0.84(0.53 - 1.32) |

**Note: APR: Adjusted prevalence ratio ***Significant at p-value < 0.001. **Significant at p-value < 0.01. *Significant at p-value < 0.05.**

**Mean Number of NCDs Risk Factors and Multivariable Analysis of Clustering of NCDs Risk Factors**

We investigated the clustering of NCD risk factors in our sample, ranging from 0 to 3, within individuals. We used a multivariable Poisson regression model with robust standard error. **Table 4** presents that women aged 40-49 years and 30-39 years were, on average three folds (ARR:3.19; 95% CI:2.68-3.80) and twice (ARR: 2.24; 95% CI:1.90-2.64) likely to experience multiple NCDs risk factors relative to the younger age group of women. In comparison with the never-married women, married (ARR:1.91; 95% CI:1.49-2.45) and widowed/divorced/separated women (ARR:2.25; 95% CI:1.64-3.08) were more likely to multiple NCDs risk factors. Individuals in Koshi(ARR:1.74; 95% CI:1.41-2.15), Bagmati(ARR:1.71; 95% CI:1.38-2.11), Gandaki(ARR:1.65; 95% CI:1.33 - 2.06), Lumbini(ARR:1.56, 95% CI:1.26-1.92), and Karnali provinces(ARR:1.55; 95% CI:1.24 - 1.94) were staying with a somewhat higher number of NCDs risk factors on average compared to the individuals in Sudurpaschim province. Similarly, women belonging to the richer (ARR:1.56, 95% CI:1.31-1.84) and richest wealth quintile (ARR:1.5; 95% CI: 1.24 - 1.85) were more likely to have multiple risk factors of NCDs on average relative to women from the poorest wealth quintile. Women who were on service work (ARR:1.23; 95% CI: 1.06-1.43) were also more likely to experience multiple NCD risk factors compared to women not on work. In contrast, women speaking Maithali (ARR:0.54; 95% CI:0.41-0.70) and Bhojpuri (ARR:0.39; 95% CI:0.26-0.59) as native languages were less likely to experience multiple NCDs risk factors on average relative to Nepali native speaker women.

**Table 4: Mean Number of NCD Risk Factors and Multivariable Analysis (Poisson regression model with robust sandwich variance estimates) of Clustering of NCD Risk Factors.**

| **Variables** | **Mean Number (SD)** | **Clustering of NCD Risk Factors, ARR (95% CI)** |
| --- | --- | --- |
| **Total** | 0.43(0.63) |  |
| **Place of Residence** |  |  |
| Urban | 0.46(0.64) | 1.05(0.94 - 1.17) |
| Rural | 0.36(0.60) | Reference |
| **Province** |  |  |
| Koshi | 0.45(0.64) | 1.74(1.41 - 2.15) *** |
| Madhesh | 0.25(0.51) | 1.6(1.19 - 2.13) ** |
| Bagmati | 0.61(0.70) | 1.71(1.38 - 2.11) *** |
| Gandaki | 0.56(0.67) | 1.65(1.33 - 2.06) *** |
| Lumbini | 0.41(0.62) | 1.56(1.26 - 1.92) *** |
| Karnali | 0.38(0.59) | 1.55(1.24 - 1.94) *** |
| Sudurpaschim | 0.24(0.47) | Reference |
| **Native Language** |  |  |
| Nepali | 0.49(0.65) | 2.57(1.69 - 3.90) *** |
| Maithali | 0.22(0.47) | 1.38(0.89 - 2.11) |
| Bhojpuri | 0.15(0.36) | Reference |
| Other | 0.35(0.57) | 2.62(1.63-4.21) *** |
| **Marital Status** |  |  |
| Never Married | 0.14(0.41) | Reference |
| Married or Living together | 0.50(0.64) | 1.91(1.49 - 2.45) *** |
| Widow/Divorced/Separated | 0.80(0.80) | 2.25(1.64 - 3.08) *** |
| **Women's Occupational Status** |  |  |
| Not Working | 0.33().56) | Reference |
| Services | 0.64(0.68) | 1.23(1.06 - 1.43) ** |
| Agriculture-Self-employed | 0.39(0.61) | 0.99(0.86 - 1.14) |
| Manual | 0.52(0.71) | 1.14(0.94 - 1.38) |
| **Women's Education** |  |  |
| No education | 0.53(0.69) | 1.05(0.87 - 1.26) |
| Primary | 0.41(0.62) | 0.98(0.83 - 1.16) |
| Some secondary | 0.35(0.57) | 0.93(0.79 - 1.09) |
| SLC and above | 0.41(0.60) | Reference- |
| **Women's Age** |  |  |
| 15-29 yrs | 0.18(0.42) | Reference |
| 30-39 yrs | 0.58(0.64) | 2.24(1.90 - 2.64) *** |
| 40-49 | 0.80(0.76) | 3.19(2.68 - 3.80) *** |
| **Caste/Ethnicity** |  |  |
| Brahmin Hill | 0.45(0.62) | Reference |
| Chhetri Hill | 0.41(0.60) | 1.13(0.93 - 1.36) |
| Terai Caste | 0.26(0.51) | 1.06(0.81 - 1.37) |
| Dalit | 0.41(0.63) | 1.42(1.15 - 1.75) ** |
| Hill Janajati | 0.61(0.70) | 1.44(1.21 - 1.72) *** |
| Terai Janajati | 0.28(0.54) | 0.84(0.64 - 1.09) |
| Muslim | 0.31(0.54) | 1.24(0.88 - 1.74) |
| **Wealth Index Combined** |  |  |
| Poorest | 0.33(0.56) | Reference |
| Poorer | 0.32(0.56) | 1.06(0.89 - 1.25) |
| Middle | 0.33(0.57) | 1.15(0.96 - 1.38) |
| Richer | 0.52(0.68) | 1.56(1.31 - 1.84) *** |
| Richest | 0.60(0.68) | 1.52(1.24 - 1.85) *** |

**Note: ARR: Adjusted Risk Ratio, SD: Standard Deviation; ***Significant at p-value < 0.001. **Significant at p-value < 0.01. *Significant at p-value < 0.05.**

**DISCUSSION**

In our findings, we observed that the prevalence of smoking, overweight/obesity, and hypertension were 3.9%, 29.2%, and 9.6%, respectively. These prevalence figures demonstrate a relatively higher prevalence of NCD risk factors within the studied population. Our findings are consistent with past studies from LMICs, which have shown growth in NCD risk factors burden among women of reproductive age 15-49 years (53). Potential underlying reasons behind the increase in NCD risk factors could be unhealthy food habits, physical inactivity, and economic growth, leading to nonessential and junk food expenses (54). However, it’s important to note that the distribution of these established NCD risk factors varies across different explanatory variables.

**Current Smoking**

Our study found a high prevalence of smoking among women who were aged 40+ years, illiterate, engaged in agriculture/manual work, and from poor socioeconomic status. More than three-fold prevalence of smoking among those aged 40-49 years has been consistent with another study (11, 14, 55). Older women might have misunderstandings, such as thinking that quitting smoking later in life offers no advantages or that smoking a small number of cigarettes carries no adverse health effects (56). The prevalence of current smoking (3.9%) has declined significantly from the previous round of NDHS (6.0%), with a steep decline in Sudurpaschim province. This decline is consistent with reports from the WHO, which estimated a sharp decline from 8.9%(2000) to 1.6%(2025) among women in the South East Asia Regional Office (57). This could be due to the implementation of a multisectoral action plan for the prevention and control of NCDs, increasing awareness among relatively younger reproductive-age women of the harmful effects of smoking, and strict enforcement of directives for the packaging of tobacco products. Our findings were similar to those of previous studies in Nepal (14) and Bangladesh (11).

**Hypertension & Overweight/Obesity**

Over one in ten reproductive-aged women in Koshi, Gandaki, and Bagmati provinces were hypertensive in 2022. The prevalence of hypertension was significantly higher among women from the older age group and Koshi province relative to the younger age group and Sudurpaschim province, respectively. Certain factors, including high salt intake, low consumption of fruits and vegetables, physical inactivity, and obesity, as well as emerging risk factors like air pollution, urbanization, and the depletion of green spaces, may contribute to elevated hypertension (58).

The study also found a higher prevalence of overweight and obesity among women residing in Bagmati and Gandaki province, those who are married, widowed/divorced/separated, and women employed in the service sector, as well as among older women, disadvantaged Dalit and hill Janajati groups, and relatively wealthier segments of the population. The overall high prevalence of obesity might be a contributing factor behind the rise in hypertension between 2016 and 2022. Overall, 75% of the incidence of hypertension is estimated to be associated with overweight and obesity, irrespective of gender(59). However, obesity might not be a contributing factor behind the increase in hypertension among women from Koshi province, and there might be other underlying factors that need to be further investigated. A potential explanation beyond obesity behind increasing hypertension could be the worsening air quality of Nepal over the years, as it has been established as an independent risk factor for high blood pressure (60). The air quality of Nepal is unhealthy and 8.5 times more polluted compared to the WHO annual air quality value (61), and the situation, especially in the capital city, has worsened over the years (62).

**Clustering of NCD Risk Factors**

Clustering of NCD risk factors appears to be more prevalent among older individuals, those from wealthier segments of the population, disadvantaged Dalits and Janajatis, as well as among married, widowed, divorced, or separated individuals, and women employed in the service sector. We found that about 6.6% of the study participants reported having double risk factors, consistent with the earlier study, which reported 6.5% (14). We observed that the clustering of NCD risk factors increased with age and wealth index. This finding aligns with the increasing NCD risk factors identified in previous studies from various countries, including Nepal (11, 14, 63). The clustering of NCD risk factors among richer/richest women could be attributed to a sedentary lifestyle. This lifestyle adoption may also explain the relatively higher clustering of NCD risk factors observed across different provinces, such as Koshi, Bagmati, and Gandaki.

The observed province-wise trend in risk factor clustering aligns with the findings of previous studies in Nepal (14). Furthermore, the multidimensional poverty rate in these three provinces, as indicated by an economic survey conducted in 2018-2019, is below the national average (33). This lower poverty rate could have facilitated urbanization and changes in dietary patterns, thereby contributing to the persistent clustering of risk factors among women in specific provinces over time. Similarly, the higher prevalence of clustered risk factors among Dalit and Hill Janajati groups might be attributed to intertwined factors such as wealth inequalities, residence in remote areas, lower educational attainment, and certain lifestyle practices (64, 65), consistent with other studies (14).

**Implications for Policy and Programs**

The increasing prevalence of smoking among older women indicates the necessity for targeted behavioural intervention aimed at addressing specific misconceptions among older female smokers. Moreover, effective enforcement of tobacco control policies is essential. This may include developing tobacco cessation mobile applications, prioritizing tobacco cessation programs at the PHC level, and formulating national guidelines for cessation services. Additionally, continuous monitoring and compliance with tobacco control policies from multiple stakeholders, along with raising taxes on tobacco products, can be cost-effective measures to reduce smoking rates among older women of reproductive age (25, 66). Similarly, there is an urgent need to integrate NCD prevention, screening, diagnosis, and management into widely available maternal newborn and child health (MNCH) to prevent unfavourable maternal and newborn outcomes (67, 68). Despite the increasing prevalence of women’s NCD risk factors, access to NCD services remains limited, especially in low-resource settings, due to gender inequalities, social norms prioritizing maternal and child health needs, and poor awareness of NCDs and their risk factors, particularly in rural areas (68, 69). The inadequacy of health system readiness further exacerbates these challenges, marked by a shortage of trained staff, guidelines, and diagnostic equipment for NCD services (28, 70). While health system policies acknowledge the growing burden of NCDs, they fall short of effectively addressing NCD risk factors (29). Leveraging community health workers and volunteers in rural communities of Nepal could be a viable solution to increase awareness, early detection, and management of NCD risk factors among women in low-resource settings (71, 72). Moreover, implementing policies and programs that specifically target women from wealthier families, Dalit and Hill Janajati communities, older women, and women in service can help prevent the clustering of NCD risk factors among reproductive-aged women (15-49 years).

**Strength and Limitations**

A major strength of this study was the use of recent population-based, nationally representative data sources. The NDHS covered all seven provinces' rural and urban areas, making these findings generalizable to country and province levels. Modified Poisson regression was used for analysis to correct the poor estimates of association produced by conventional logistic regression and improve the estimates’ precision. However, this study also had some limitations. Firstly, since it was a cross-sectional study, the study did not establish causality. A prospective cohort study may be beneficial in determining the longitudinal progression of NCD risk factors from single to successive concurrence. Secondly, NDHS did not record other crucial clinical biomarkers (e.g., blood lipid profiles, HbA1c, serum creatinine), as well as behavioural factors (e.g., alcohol consumption, sleep duration), dietary factors (e.g., type and amount of food taken), physical activity, which are important risk factors for NCDs. So, this information could not be included in the analysis, which limits the strength of this study. Thirdly, the reliance on self-reported data for current smoking status presents another limitation. There might be the possibility of underreporting due to social stigma, particularly among younger women, which could have introduced misclassification bias into findings, leading to underestimation of prevalence rates.

**CONCLUSION**

This study found several markers of NCD risk factors clustering among women of reproductive age in Nepal. To address this, an urgent focus is needed to prioritize increasing awareness, screening, and management of NCD risk factors among women of reproductive age. Failure to address this can increase the risk of adverse outcomes, undermining efforts to achieve the sustainable development goal of reducing premature deaths from NCD by one-third by 2030.

**Declarations**

**Funding**
None

**Data availability**

The study used publicly available data from the DHS surveys. More information about data access is available elsewhere. https://dhsprogram.com/data/available-datasets.cfm

**Acknowledgement**

The authors would like to acknowledge the Demographic and Health Survey (DHS) program for granting access to the data of Nepal Demographic and Health Survey NDHS) 2016 and 2022

**Authors contribution**

**Barun Kumar Singh**, Conceptualization, Data curation, Formal analysis, Writing-original draft, Writing-review, and editing^1^

**Shiva Raj Mishra**, Supervision, Validation, Writing-review & editing^2,3^

**Resham B Khatri,** Conceptualization, Supervision, Validation, Writing- review & editing^4^

**REFERENCES**

1.	World Health Organization(WHO). Noncommunicable Diseases: WHO; 2023 [cited 2024 7 May 2024 ]. Available from: <https://www.who.int/news-room/fact-sheets/detail/noncommunicable-diseases>.

2.	World Health Organization(WHO). Non Communicable Diseases Progress Monitor 2022: World Health Organization(WHO); 2022. Available from: <https://www.who.int/publications/i/item/9789240047761>.

3.	Dhungana RR, Karki KB, Bista B, Pandey AR, Dhimal M, Maskey MK. Prevalence, pattern and determinants of chronic disease multimorbidity in Nepal: secondary analysis of a national survey. BMJ Open. 2021;11(7):e047665.

4.	Aryal K, Neupane S, Mehata S, Vaidya A, Singh S, Paulin P, et al. Non communicable diseases risk factors: steps survey Nepal. Kathmandu: Nepal Health Research Council. 2013.

5.	World Health Organisation. Stepwise approach to chronic disease risk factor surveillance: 2007 steps survey report. 2008.

6.	McCracken K, Phillips DR. Demographic and Epidemiological Transition. International Encyclopedia of Geography2017. p. 1-8.

7.	Pandey AR, Chalise B, Shrestha N, Ojha B, Maskey J, Sharma D, et al. Mortality and risk factors of disease in Nepal: Trend and projections from 1990 to 2040. PLoS One. 2020;15(12):e0243055.

8.	Budreviciute A, Damiati S, Sabir DK, Onder K, Schuller-Goetzburg P, Plakys G, et al. Management and Prevention Strategies for Non-communicable Diseases (NCDs) and Their Risk Factors. Front Public Health. 2020;8:574111.

9.	Darmansjah D, Kalra G, Bhugra D, Bhugra D, Ventriglio A, Castaldelli-Maia J, et al. 49Globalization and urbanization. In: Bhugra D, Ventriglio A, Castaldelli-Maia J, McCay L, editors. Urban Mental Health: Oxford University Press; 2019. p. 0.

10.	Mishra SR, Neupane D, Bhandari PM, Khanal V, Kallestrup P. Burgeoning burden of non-communicable diseases in Nepal: a scoping review. Globalization and Health. 2015;11(1):32.

11.	Chowdhury SR, Islam MN, Sheekha TA, Kader SB, Hossain A. Prevalence and determinants of non-communicable diseases risk factors among reproductive-aged women: Findings from a nationwide survey in Bangladesh. PLOS ONE. 2023;18(6):e0273128.

12.	Khuwaja AK, Kadir MM. Gender differences and clustering pattern of behavioural risk factors for chronic non-communicable diseases: community-based study from a developing country. Chronic Illness. 2010;6(3):163-70.

13.	Gluckman PD, Hanson MA, Bateson P, Beedle AS, Law CM, Bhutta ZA, et al. Towards a new developmental synthesis: adaptive developmental plasticity and human disease. The Lancet. 2009;373(9675):1654-7.

14.	Bista B, Dhungana RR, Chalise B, Pandey AR. Prevalence and determinants of non-communicable diseases risk factors among reproductive aged women of Nepal: Results from Nepal Demographic Health Survey 2016. PLoS One. 2020;15(3):e0218840.

15.	Nilsson PM, Viigimaa M, Giwercman A, Cifkova R. Hypertension and Reproduction. Current Hypertension Reports. 2020;22(4):29.

16.	Ahmed I, Ali SM, Amenga-Etego S, Ariff S, Bahl R, Baqui AH, et al. Population-based rates, timing, and causes of maternal deaths, stillbirths, and neonatal deaths in south Asia and sub-Saharan Africa: a multi-country prospective cohort study. The Lancet Global Health. 2018;6(12):e1297-e308.

17.	Hu FB. Overweight and obesity in women: health risks and consequences. J Womens Health (Larchmt). 2003;12(2):163-72.

18.	Asogwa OA, Boateng D, Marzà-Florensa A, Peters S, Levitt N, Olmen Jv, et al. Multimorbidity of non-communicable diseases in low-income and middle-income countries: a systematic review and meta-analysis. BMJ Open. 2022;12(1):e049133.

19.	Kapur A, Hod M. Maternal health and non-communicable disease prevention: An investment case for the post COVID-19 world and need for better health economic data. Int J Gynaecol Obstet. 2020;150(2):151-8.

20.	Fabienke R, Hod M, Kapur A. Take home message: Start where life begins and follow the life-course approach. Diabetes Res Clin Pract. 2018;145:214-5.

21.	Health Mo, Population , ICF. Nepal Demographic and Health Survey 2022. Kathmandu, Nepal: Ministry of Health and Population [Nepal]; 2023.

22.	Ministry of Health and Population(MoHP). Multisectoral Action Plan for the Prevention and Contol of Non Communicable Diseases(2014-2020). Kathmandu: Government of Nepal; 2014.

23.	Ministry of Health and Population(MoHP). Multisectoral action plan for the prevention and control of non-communicable diseases(2021-2025). Kathmandu: Government of Nepal; 2021.

24.	Tobacco Product(Control and Regulatory) Act, (2011).

25.	Khanal GN, Khatri RB. Burden, prevention and control of tobacco consumption in Nepal: a narrative review of existing evidence. Int Health. 2021;13(2):110-21.

26.	Ministry of Health and Population(MoHP). Annual Health Report 2079/2080. Kathmandu: Government of Nepal; 2024. p. 326.

27.	Gyawali B, Khanal P, Mishra SR, van Teijlingen E, Wolf Meyrowitsch D. Building Strong Primary Health Care to Tackle the Growing Burden of Non-Communicable Diseases in Nepal. Glob Health Action. 2020;13(1):1788262.

28.	Adhikari B, Pandey AR, Lamichhane B, KC SP, Joshi D, Regmi S, et al. Readiness of health facilities to provide services related to non-communicable diseases in Nepal: evidence from nationally representative Nepal Health Facility Survey 2021. BMJ Open. 2023;13(7):e072673.

29.	Sapkota BP, Baral KP, Berger U, Parhofer KG, Rehfuess EA. Health sector readiness for the prevention and control of non-communicable diseases: A multi-method qualitative assessment in Nepal. PLoS One. 2022;17(9):e0272361.

30.	Rahimi E, Mohammadi R, Mokhayeri Y, Nazari SS. Gender disparity and risk of noncommunicable disease among adults in Islamic Republic of Iran. East Mediterr Health J. 2023;29(8):630-7.

31.	Adhikari K, Gupta N, Koshy AK. Gender differences on risk factors of non-communicable diseases--a community based cross-sectional study in central Nepal. J Nepal Health Res Counc. 2014;12(27):89-93.

32.	DeVon HA, Ryan CJ, Ochs AL, Shapiro M. Symptoms across the continuum of acute coronary syndromes: differences between women and men. Am J Crit Care. 2008;17(1):14-24; quiz 5.

33.	Gyawali B, Mishra SR, Ghimire S, Hansen MRH, Shah KJ, Subedee KC, et al. The burden and correlates of multiple cardiometabolic risk factors in a semi-urban population of Nepal: a community-based cross-sectional study. Scientific Reports. 2019;9(1):15382.

34.	Bista B, Dhimal M, Bhattarai S, Neupane T, Xu YY, Pandey AR, et al. Prevalence of non-communicable diseases risk factors and their determinants: Results from STEPS survey 2019, Nepal. PLoS One. 2021;16(7):e0253605.

35.	Dahal S, Sah RB, Niraula SR, Karkee R, Chakravartty A. Prevalence and determinants of non-communicable disease risk factors among adult population of Kathmandu. PLOS ONE. 2021;16(9):e0257037.

36.	Shrestha N, Karki K, Poudyal A, Aryal KK, Mahato NK, Gautam N, et al. Prevalence of diabetes mellitus and associated risk factors in Nepal: findings from a nationwide population-based survey. BMJ Open. 2022;12(2):e060750.

37.	Hasan M, Sutradhar I, Akter T, Das Gupta R, Joshi H, Haider MR, et al. Prevalence and determinants of hypertension among adult population in Nepal: Data from Nepal Demographic and Health Survey 2016. PLOS ONE. 2018;13(5):e0198028.

38.	Dhungana RR, Pandey AR, Bista B, Joshi S, Devkota S. Prevalence and Associated Factors of Hypertension: A Community-Based Cross-Sectional Study in Municipalities of Kathmandu, Nepal. International Journal of Hypertension. 2016;2016:1656938.

39.	Mehata S, Shrestha N, Mehta R, Vaidya A, Rawal LB, Bhattarai N, et al. Prevalence, awareness, treatment and control of hypertension in Nepal: data from nationally representative population-based cross-sectional study. Journal of Hypertension. 2018;36(8).

40.	Tandon K, Adhikari N, Adhikari B, Pradhan PMS. Co-occurrence of non-communicable disease risk factors and its determinants among school-going adolescents of Kathmandu Metropolitan City. PLoS One. 2022;17(8):e0272266.

41.	Piryani S, Baral KP, Pradhan B, Poudyal AK, Piryani RM. Overweight and its associated risk factors among urban school adolescents in Nepal: a cross-sectional study. BMJ Open. 2016;6(5):e010335.

42.	Ministry of Health - MOH/Nepal, New ERA/Nepal, ICF. Nepal Demographic and Health Survey 2016. Kathmandu, Nepal: MOH/Nepal, New ERA, and ICF; 2017.

43.	ICF International. MEASURE DHS biomarker field manual. Calverton, Maryland, USA: ICF International; 2021. Available from: <https://dhsprogram.com/pubs/pdf/DHSM7/DHS8_Biomarker_Manual_English_27Sep2021.pdf>.

44.	Chobanian AV, Bakris GL, Black HR, Cushman WC, Green LA, Izzo JL, Jr., et al. The Seventh Report of the Joint National Committee on Prevention, Detection, Evaluation, and Treatment of High Blood Pressure: the JNC 7 report. Jama. 2003;289(19):2560-72.

45.	Whitworth JA. 2003 World Health Organization (WHO)/International Society of Hypertension (ISH) statement on management of hypertension. J Hypertens. 2003;21(11):1983-92.

46.	Rutstein SO, Johnson K. The DHS wealth index. Calverton, Maryland, USA: ORC Macro; 2004.

47.	Barros AJD, Hirakata VN. Alternatives for logistic regression in cross-sectional studies: an empirical comparison of models that directly estimate the prevalence ratio. BMC Medical Research Methodology. 2003;3(1):21.

48.	Tamhane AR, Westfall AO, Burkholder GA, Cutter GR. Prevalence odds ratio versus prevalence ratio: choice comes with consequences. Stat Med. 2016;35(30):5730-5.

49.	Martinez BAF, Leotti VB, Silva GdSe, Nunes LN, Machado G, Corbellini LG. Odds Ratio or Prevalence Ratio? An Overview of Reported Statistical Methods and Appropriateness of Interpretations in Cross-sectional Studies with Dichotomous Outcomes in Veterinary Medicine. Frontiers in Veterinary Science. 2017;4.

50.	Chen W, Qian L, Shi J, Franklin M. Comparing performance between log-binomial and robust Poisson regression models for estimating risk ratios under model misspecification. BMC Medical Research Methodology. 2018;18(1):63.

51.	Zou G. A modified poisson regression approach to prospective studies with binary data. Am J Epidemiol. 2004;159(7):702-6.

52.	Heeringa SG, West BT, Berglund PA. Applied survey data analysis: chapman and hall/CRC; 2017.

53.	Ali M, Amin MR, Jarl J, Saha S. Prevalence, trends, and inequality in noncommunicable diseases in Bangladesh: Evidence from Bangladesh Demographic and Health Surveys 2011 and 2017–2018. Public Health Challenges. 2024;3(1):e148.

54.	Ford ND, Patel SA, Narayan KM. Obesity in Low- and Middle-Income Countries: Burden, Drivers, and Emerging Challenges. Annu Rev Public Health. 2017;38:145-64.

55.	Sreeramareddy CT, Pradhan PM, Sin S. Prevalence, distribution, and social determinants of tobacco use in 30 sub-Saharan African countries. BMC Medicine. 2014;12(1):243.

56.	Donzé J, Ruffieux C, Cornuz J. Determinants of smoking and cessation in older women. Age Ageing. 2007;36(1):53-7.

57.	World Health Organisation. WHO global report on trends in prevalence of tobacco use 2000-2025, third edition: World Health Organization; 2019 [cited 2024 9 May 2024]. Available from: <https://iris.who.int/bitstream/handle/10665/330221/9789240000032-eng.pdf?sequence=1>.

58.	Schutte AE, Srinivasapura Venkateshmurthy N, Mohan S, Prabhakaran D. Hypertension in Low- and Middle-Income Countries. Circ Res. 2021;128(7):808-26.

59.	Leggio M, Lombardi M, Caldarone E, Severi P, D'Emidio S, Armeni M, et al. The relationship between obesity and hypertension: an updated comprehensive overview on vicious twins. Hypertension Research. 2017;40(12):947-63.

60.	Giorgini P, Di Giosia P, Grassi D, Rubenfire M, Brook RD, Ferri C. Air Pollution Exposure and Blood Pressure: An Updated Review of the Literature. Curr Pharm Des. 2016;22(1):28-51.

61.	IQAir. World Air Quality Report Staad: IQAir, ; 2023 [cited 2024 9 May 2024]. Available from: <https://www.iqair.com/world-air-quality-report>.

62.	Paudel A. Air quality reaches ‘unhealthy’ levels in Kathmandu Valley, sparking public health concerns. Kahtmandu Post. 2023 7 November 2023.

63.	Biswas T, Townsend N, Islam MS, Islam MR, Gupta RD, Das SK, et al. Association between socioeconomic status and prevalence of non-communicable diseases risk factors and comorbidities in Bangladesh: findings from a nationwide cross-sectional survey. BMJ Open. 2019;9(3):e025538.

64.	Dhimal M, Bista B, Bhattarai S, Dixit L, Hyder M, Agrawal N, et al. Report of Non Communicable Disease Risk Factors: STEPS Survey Nepal 2019. Kathmandu: Nepal Health Research Council; 2020 [cited 2024 9 May 2024]. Available from: <https://www.who.int/docs/default-source/nepal-documents/ncds/ncd-steps-survey-2019-compressed.pdf>.

65.	Pandey JP, Dhakal MR, Karki S, Poudel P, Pradhan MS. Maternal and Child Health in Nepal: The Effects of Caste, Ethnicity, and Regional Identity: Further analysis of the 2011 Nepal Demographic and Health Survey. Calverton, Maryland, USA: Ministry of Health and Population, New ERA, and ICF International; 2013 [cited 2024 19 May 2024]. Available from: <https://www.dhsprogram.com/pubs/pdf/FA73/FA73.pdf>.

66.	Shrestha I, Shakya S, Hatch B. Enforcement of Tobacco Control Policies in Nepal2021.

67.	Akselrod S, Banerjee A, Collins TE, Acharya S, Artykova N, Askew I, et al. Integrating maternal, newborn, child health and non-communicable disease care in the sustainable development goal era. Front Public Health. 2023;11:1183712.

68.	Alliance N. Women and NCDs: NCD Alliance; 2023 [updated 8/3/2023; cited 2024 15 May 2024]. Available from: <https://ncdalliance.org/why-ncds/ncds-and-sustainable-development/women-and-ncds>.

69.	Singh SK, Chauhan K, Puri P. Chronic non-communicable disease burden among reproductive-age women in India: evidence from recent demographic and health survey. BMC Women's Health. 2023;23(1):20.

70.	Thapa DK, Acharya K, Karki A, Cleary M. Health facility readiness to provide antenatal care (ANC) and non-communicable disease (NCD) services in Nepal and Bangladesh: Analysis of facility-based surveys. PLoS One. 2023;18(3):e0281357.

71.	Safary E, Mwandeti M, Matanje B, Beiersmann C, Mtaita C, Shiroya V, et al. Role of community health volunteers in identifying people with elevated blood pressure for diagnosis and monitoring of hypertension in Malawi: a qualitative study. BMC Cardiovasc Disord. 2021;21(1):361.

72.	Mishra SR, Neupane D, Preen D, Kallestrup P, Perry HB. Mitigation of non-communicable diseases in developing countries with community health workers. Globalization and Health. 2015;11(1):43.
