# Supplementary material for: Trends and determinants of clustering for non-communicable disease risk factors in women of reproductive age in Nepal
Source: PLoS One. 2024 Oct 2;19(10):e0309322. doi: 10.1371/journal.pone.0309322 (PMC11446422; doi:10.1371/journal.pone.0309322)
Supplement: Attachment — Submitted filename: Response to Reviewers.docx [file pone.0309322.s004.docx]

**Point-by-point responses to editor and reviewer’s’ comments**

| **Comments** | **Responses** |
| --- | --- |
| **Editor’s comments** |  |
| What about other factors for NCDs | Thanks a lot for query. As one of our objectives was to estimate the trend of prevalence of NCD risk factor (smoking, overweight/obesity and hypertension) between 2016 and 2022, we could not include alcohol, as it was not measured in 2016 NDHS. However there are other risk factors such as physical activities, fruit/vegetable intake, and some biomarkers, that were not available in NDHS, and for that purpose we need to rely on STEPS survey, that was beyond the scope of our study. |
| It is unclear, what is total sample size? What is sample size for 2016? Why hypertension included only in 2022? Please clarify the sample size and reason for choosing women only and adjsutement for different variables? | Thanks a lot for query. We performed the analysis separately as per our objective of the paper, and did not pool the data from two round of NDHS survey (2016 and 2022). Our analytical sample size was different depending on the outcomes (smoking, hypertension, overweight/obesity) and we applied same inclusion and exclusion criteria for both 2016 and 2022 (excluding currently pregnant women and mother up to 2 months). We have revised and included in the abstract. |
| why did you poisson model and not other models? | To avoid the overestimation of association by odds ratio in cross-sectional studies, the prevalence ratio for reporting the association was used. |
| If author follows 2021 census this death rate is 98,736 deaths were due to NCDs, This number is difference ? clarify and link it with census data .https://thehimalayantimes.com/nepal/non-communicable-diseases-responsible-for-71-per-cent-deaths-in-nepal | Thanks a lot for your insightful comments, and providing the source of updated figures. Now we have changed accordingly in the introduction section. |
| Does it mean maternal deaths associated with NCDS? If yes, how. It is better to include in first paragraph | Thanks a lot for your valuable feedback. This has been included and revised. As we talk about epidemiological transition, obstetric transition is happening concurrently in Nepal which is characterised by moving away from high maternal death attributable to direct cause to maternal deaths attributed to indirect causes, and that is particularly aggravated by pre-existing conditions such as NCDs. We have provided the plausible reason behind the association of maternal deaths attributable to NCD conditions and risk factors in text based on your feedback. Further we have discussed about the NCD risk factors among overall population and then discussed about (the NCD risk factors) among women. We are grateful for your comments and opportunity to revise. |
| Is there any factors besides hypertention ? If yes describe it ? | Thanks a lot for comments and suggestion. Now we have added the other factors besides hypertension in that list such as chronic stress, and increasing childbearing age. Unfortunately, these variables were not of interest in this study, we have not gone particularly in details about explaining these underlying risk factors in this study as per our objectives. |
| What are the outcome of these programs? | Thanks a lot for comments. Now we have added the outcome of those program documented in recent literature. |
| To whom and how? Better to mention outcome of success | Thanks a lot for your inputs. MSAP (2^nd^ phase-2021-2025) for NCD is ongoing in Nepal, some evidence have been documented in recent article based on Nepal health facility survey and some qualitative analysis. We have added those evidence in introduction section in page 4. |
| To Whom? Nepali women or global | Thanks a lot for pointing out error/typo. Now we have added explicitly in which context, that statement applies. |
| Gap has mentioned about diabetics and hypertension. Nothing is mentioned about it in aim of the study. | Actually, we have mentioned the gap in context of standalone risk factor, previous study has reported. Our main aim of the study was to report as clustering of risk factor and then describe the predictors associated with these clustered risk factors. |
| How did you extract variables from large amount of variables as data are coded and decoded? Please describe it in method section. | Now we have revised data sources and study population information including explanation of explanatory variables. |
| What ate clustered risk factors? | Clustered risk factor means if someone possesses a multiple risk factor, and there is tendency for person to suffer simultaneously from behavioural and metabolic risk factors, as these are highly correlated. |
| Authors had pulled the data between two NDHS? Please mention how did you combined data set ? | Response: Sorry for the misunderstanding, and thanks for raising your concern. We did not pool the data from NDHS 2016 and 2022; we performed the analysis separately. Our prime aim of the study was to evaluate the determinants of clustered NCD risk factors among women of reproductive age based on the latest NDHS 2022 dataset. However, the second objective was to compare the trend of prevalence of individual NCD risk factors (obesity, hypertension, smoking status) at the geographical level (province and national) based on the last two surveys, which we performed separate analyses and plotted on the appropriate graph showing trend (Figure 1). For your reference, we have also uploaded *stata* do file into the system. |
|  |  |
| Is there any difference between modified Poisson and Poisson (see abstract) as both terms were used? | Thanks a lot for pointing out the inconsistency, now we have corrected the abstract section by replacing it with “modified Poisson”. |
| Did you plan to include do file in this manuscript? if not , justify its reason. | Thanks a lot for this query. We don’t have any objection to upload *stata* do file in the portal. We have uploaded the *stata* do file for your perusal. |
| Unable to find figure 1 | Thanks a lot for comment. Sorry we missed uploading figure 1. Now figure 1 has been upload along with the manuscript. |
| Can you please Clarify it ? Did you mean 2016 data? If yes, define properly. Did you combine or separate data or pooled analysis discuss in method section? | Thanks a lot for your critical comment**.** we analysed 2016 and 2022 data to meet our first objective to evaluate the trend of each risk factor at national and provincial level, so that, our province will be benefitted from comparison. We didn’t pool the data together and did analysis. Rather we applied independent sample proportion (Wald-z test), assuming equal variance in risk factor at both survey on the prevalence figure derived from separate analysis in STATA by using command *prtesti* |
| Please bold significant results for clarity | Thanks a lot for comment. Now we have made the significant p-value bold. |
| Do not repeat all results from table ? | Thanks a lot for your input. Now we have made the changes in description of result and kept important one. |
| Elaborate it in methodology. Why alcohol is not included ? | Thanks a lot for insightful comment. The study team didn’t take into account harmful use of alcohol in this study, as it was measured first time in latest NDHS 2022 and was missing in NDHS 2016. Eventually, one of our objectives was to compare the trend of prevalence of risk factors between two surveys. We have mentioned this as our limitation in Limitation section in page 20, and also written about the scope of future studies. |
| See previous commend and make terminology uniform | Thanks a lot. Now we have made most the terminologies consistent. |
| Sd exceed mean ? mean risk factor is below 1. Can you interpret its meaning prior to ARR? | Thanks a lot for your critical comment. As we know that, under assumption of poisson regression, mean and variance for random variable are equal, if we square the standard deviation, variance is around mean. Obviously in some cases, this assumption is violated and that was one of the reasons to choose the modified poisson regression with robust sandwich variance estimate. Moreover, mean risk factor is below 1, as this data is heavily weighted by lots of ‘zeors’ among those who do not have any NCD risk factors (approx. 64%). That’s why we didn’t interpret the mean risk factor and focused on interpreting adjusted risk ratio (ARR=i.e. ratio of count), which would be meaningful. Thank you once again for raising valid point. |
| Is the data of your study and reference 14 is same ? if yes, we cannot include as similar findings. | Thanks a lot for your query. No, the data of our study is NDHS 2022, whereas study of reference 14 is based on NDHS 2016. |
| Everybody explains about intervention but what types of intervention is essential to reduce intervention | Thanks a lot for your remarks. We have listed the interventions required to address the issue of relatively higher prevalence of smoking among older women. |
| This is important in present context. How it can implemented in essential than just mention its need | Thanks a lot for your insightful comments**.** We also believe that health and demographic surveillance system (HDSS) should be established with sustainable funding as it has been successful in another parts of world such as Bangladesh (MATLAB) and Ghana. We have added the establishment of HDSS as a way to cover longitudinal progression of risk factor in our country with appropriate citation. |
| Though the data are available from DHS data base. It is important to mention what types of data extracted and its availability (current study) and to do included in Annex part of this study. | Thanks a lot for valuable inputs. We have updated the information in that section of data availability about the data sets used for the analysis, and will also upload into system as supplementary information. |

| **Comments** | **Response** |
| --- | --- |
| **Reviewer 1** |  |
| Title: Please avoid the last part of title and just say “The patterns of clustering of non-communicable diseases risk factors among women of reproductive age in Nepal”. | **tha** Thanks a lot for the feedback, now we have removed the last part of title, and modified as per your suggestion |
| Background: Please avoid using pronoun such as we, our etc. In the last sentence of the Background, avoid We aimed and say this paper aims to assess the trends of prevalence and associated factors with smoking status, overweight/obesity, and hypertension among women of reproductive age in Nepal. | Now we have replaced “We” with “this paper/study” aim to assess the trends….in full text. |
| Methods: Replace we examined with this paper examined….. | Thanks a lot. now it has been replaced with this paper instead of we. |
| Keywords: please keep keywords in ascending order (a-z) without capitalization, separated by commas but not a semi colon. | Thank you, now keyword has been arranging in ascending order alphabetically, separated by commas but not semicolon |
| Introduction: Clarify the 41 million deaths statistics by stating complete say NCDs contribute to 41 million deaths annually worldwide; 77% of these deaths occur in Low and Middle-Income Countries (LMICs). Please Provide reference? | Thank you for input, now this sentence has been revised with the reference. |
| Please choose either obese or obesity for consistency throughout the paper. | Thanks a lot for feedback. Now it has been made consistent within the sentence. |
| Please modify the statement the National Health Education, Information, and Communication Centre (NHEICC) conducts tobacco-specific advocacy and awareness activities to “The National Health Education, Information and communication (NHEICC) leads health promotion and behaviour change communication initiatives, including tobacco-specific advocacy and awareness programs implemented through multimedia channels, public private health care facilities, media and volunteers throughout the countries”. Add references from DOHS annual report or NHEICC reports. | Thanks a lot for valuable input. Now it has been edited. |
| Include additional reference related to Health promotion after reference 27. Such as: <https://journals.sagepub.com/doi/full/10.1177/17579759221117792> <https://www.nepjol.info/index.php/jhp/article/view/40957> | Thanks a lot for suggesting the appropriate citation for this paper. We have incorporated both references. |
| Move the sentence to successfully fill this gap….., after your second last sentence and before the aims of this paper. | Thanks a lot for pointing out this gap. We have made correction in main text. |
| Methods |  |
| Please simplify the sentence - to identify the prevalence and significant determinants of NCD risk factors among women, this study used the NDHS 2022 data set. You can say “this study used the NDHS 2022 dataset only. | Thanks a lot for suggestion, now we have done accordingly |
| Please define your final model, what does it mean and how did you determine a final model? | Thanks a lot for comment. We have corrected it. Actually, it was final analytical sample for our specified outcome rather than final model, |
| Explanatory variables: What was the basis of categorization of each variable? please provide reference of each variable. If using exact categorization from NDHS 2022, please cite this report. Explain any changes made to variable categories. Please delete residence, which is duplicated. Please define each explanatory variable. | Thanks a lot for such insightful comment. Now we have edited the section of explanatory variables by providing the basis for categorization of each variable, where possible with the appropriate reference and justification. Moreover, details about recoding of variable used for the study has been provided in the texts under explanatory variables. |
| Statistical analysis: Please keep references to support your claim after the statement “Variables with a chi-square test p-value below 0.25 were included in the multivariable models”. | Thanks a lot for comment to provide the reference for this cut off value of p while proceeding from univariable analysis to multivariable analysis. |
| Start your statements with Firstly, secondly, etc, to structure your arguments. | Thanks a lot for feedback. Now we have rephrased the potential reason for using Poisson regression in form of structured arguments. |
| Please insert Figure 1 and 2 in the text. | Thanks a lot for the suggestion. I will check again the journal rules, whether we need to insert the figures into text. |
| Overall comment: This paper is well written. However, please avoid repetition, ensure consistency throughout the document. Keep line numbers in the manuscript. A language review is also required. | Thank you so much for your time and efforts in providing valuable inputs on my paper. |
|  |  |
| **Reviewer 2** |  |
| This was an interesting look into patterns of Non-Communicable Diseases risk factors Clustering Among Women of Reproductive Age in Nepal. | Thanks a lot for your kind words and comments. |
| In methods: have you considered confounding factors, if so mention them | As mentioned in the objective of the study, we are looking out for factors associated with clustering of NCD risk factor among women of reproductive age. So we don’t have specific exposure of interest and are not adjusting for confounding effects of other variables in multivariable Poisson regression model. Rather we have attempted to detect multiple significant variables simultaneously by fitting into the multivariable model. While doing this, we have checked for multicollinearity and reported the variable in the main analysis (ecological zone). |
| In discussion: use more literature for the comparisons and compare the findings in terms of the regions or countries you find literature from | Thanks a lot for your critical comments. We have tried to find the similar study in similar context with similar inclusion and exclusion criteria, so that we can compare. However, to our knowledge, there were limited studies with same population and same design. |
| **Reviewer 3** |  |
| Congratulations on writing this paper. The study analysed secondary data from NDHS 2022 to determine the prevalence of three noncommunicable diseases (NCD) risk factors in Nepalese women of reproductive age group. The study further assessed the trend in the prevalence of those risk factors from 2016 to 2022. Results showed nearly one-third of those women have at least one risk factor in Nepal. The study also reported the factors associated with the prevalence and clustering of three NCD risk factors. The overall current results will have significant impact on national policy development of developing countries. The study follows the standard guidelines for reporting cross-sectional studies. The data was analysed using appropriate statistical methods. The paper has written in standard format. However, this manuscript will be further improved for clarity and accuracy by addressing the following comments. | Thanks a lot for nice summary of the paper, and valuable inputs on our paper. We tried to address all the comments, and have provided point by point response. |
| **Title** The title needs to be corrected for grammar. In the title, diseases, factors, households are nouns being used as adjectives. Therefore, these would be singular. Is it two surveys or one survey? | Thanks a lot, we have removed ‘S’ from diseases and factors and considered it as singular as it is being used as adjective  This study finding is based mostly on NDSH 2022. We have only compared the prevalence and trend of NCD risk factor only between two surveys. For the determinants purpose, we are limited to NDHS 2022 data. |
| Abstract One of the objectives of the study was to assess the trends in the prevalence of risk factors. However, the results report only the prevalence rate. There are no conclusions about the trends of those risk factors. | Thanks a lot for insightful comment. Now we have added the part about discussing increasing trend of overweight and obesity particularly in Bagmati and Gandaki province requiring urgent attention in conclusion section. |
| Method Though readers can find the sampling strategy in the cited documents, a brief paragraph is essential here as well. A sample size of NDHS 2016 should also be stated. Similarly, please state whether the inclusion and exclusion criteria are the same for the analysed data of NDHS 2016. | Thanks a lot for this critical comment. Now we have added detailed sample strategy for NDHS 2022 explaining how we reached to final analytical sample. Additionally, sample size of 2016 for all three outcomes (smoking: n=6079-unweighted, overweight/obesity/: n= 6079 hypertension: n=6076-unweighted women of reproductive age have also been included in the method section of paper. |
| Results Table 1: The indication of * to be corrected. | Thanks a lot for pointing this error. Not it has been corrected with * indicating analytical sample size for smoking and overweight/obesity, and ** indicating analytical sample size for hypertension among reproductive aged women in NDHS 2022. |
| Table 3: Please make the reference category consistent, either 1.o or ref… | Thank you for pointing out this error, now it has been resolved and have made consistent with 1.00 |
| Please report the sample size for table 4. | Thank you, the weighted analytical sample size for table is 3,765. Now we have inserted this information with the caption in Table 4 |
| Data related to hypertension is different in table 1 and figure 1. Please correct or explain (N=3765 or 3749) | Thanks a lot for your query. Unweighted analytical sample for hypertension is n = 3,765, whereas weighted sample size for outcome hypertension is n = 3,780 in NDHS 2022. |
| Figure 2 is not clear about the sample. Is it NDHS 2022 or 2016? It is not clear how sample size is 3781, when analysed hypertensive participants were 3749 for NDHS 2022 as reported in method section. | Thanks a lot for critical comment. Now we have changed the values in figure 2, and weighted sample size for clustered risk factor is 3,765, while unweighted count is 3,749. We have mentioned the unweighted sample size n=3,749, while extracting the data from NDHS 2022, and later during analysis, weighted count has been mentioned throughout the paper. |
| Discussion The first paragraph seeks the important findings of the study as specified by the overall study objectives. | Thanks a lot for insightful comment, now we have added the important findings of beginning at the discussion section. |
| Data shows prevalence of clustering of risk factors was higher in women at service than those not working. Should authors need to interpret this finding? | Thanks a lot for critical comment. Now we have discussed the higher prevalence of NCD risk factors in working women with reference to systematic study done in similar population. |
| The method and strength statement suggests that the used sample was representative of the whole country. However, the authors' conclusion is restrictive (e.g These prevalence figures demonstrate a relatively higher prevalence of NCD risk factors within the studied population). Please correct. | Thanks a lot for valuable input. Now we have corrected that sentence by replacing with women of reproductive age with “studied population”. |
| Does this increasing prevalence hold true “The increasing prevalence of smoking among older women”. The smoking trend in smoking between 2016 and 2022 is decreasing. The data was not analysed separately for the trend in older women. Please correct or explain. | Thanks a lot for such insightful comment. We agree that we didn’t apply any test to compare the trend of smoking among older women between 2016 and 2022 NDHS. However, this statement was based on comparison between previous finding and current finding. Though we have edited the sentence and discussed the policy implication based on analysis of NDHS 2022. |
| Conclusions The first sentence is not clear. “This study found several markers of NCD risk factors clustering among women of reproductive age in Nepal”. This sentence is to be rewritten. Based on data, women of reproductive age had a clustering of NCD risk factors. | Thanks a lot for suggestion. Now we have edited that part in conclusion section as per your inputs. |
| The same comment applies to the conclusions on the trends of the risk factors. | Thanks a lot. Now we have particularly specified the trend of overweight/obesity |
| **General suggestion** Line numbers will make the review process easier to refer to the specific line. | Thank you, now we will take care of that. |
