# Supplementary material for: Trends and determinants of clustering for non-communicable disease risk factors in women of reproductive age in Nepal
Source: PLoS One. 2024 Oct 2;19(10):e0309322. doi: 10.1371/journal.pone.0309322 (PMC11446422; doi:10.1371/journal.pone.0309322)
Supplement: Attachment — Submitted filename: Point by point response 2.8.2024.docx [file pone.0309322.s005.docx]

| Comments | Response |
| --- | --- |
| With whom did the author compare hill janajatis and dalit ? (see abstract) | Thanks a lot. Our reference group was Brahmin hill women, and we have added accordingly in Abstract section. |
| Please incorporate the following response in the statistical analysis section for clarity to future readers.  Thanks a lot for your critical comment. As we know that, under assumption of poisson regression, mean and variance for random variable are equal, if we square the standard deviation, variance is around mean. Obviously in some cases, this assumption is violated and that was one of the reasons to choose the modified poisson regression with robust sandwich variance estimate. Moreover, mean risk factor is below 1, as this data is heavily weighted by lots of ‘zeors’ among those who do not have any NCD risk factors (approx. 64%). That’s why we didn’t interpret the mean risk factor and focused on interpreting adjusted risk ratio (ARR=i.e. ratio of count), which would be meaningful. Thank you once again for raising valid point. | Thanks, Now we have incorporated this responses in the statistical analysis section of paper (page 9). |

**Point by point response to editor**
